# Nanocharacterization of Soft Biological Samples in Shear Mode with Quartz Tuning Fork Probes

**DOI:** 10.3390/s120404803

**Published:** 2012-04-12

**Authors:** Jorge Otero, Laura Gonzalez, Manel Puig-Vidal

**Affiliations:** SIC-BIO, Bioelectronics and Nanobioengineering Group, Department of Electronics, University of Barcelona, Marti i Franques, 1, 08028 Barcelona, Spain; E-Mails: jotero@el.ub.es (J.O.); lgonzalez@el.ub.es (L.G.)

**Keywords:** tuning fork, atomic force microscopy, nanocharacterization

## Abstract

Quartz tuning forks are extremely good resonators and their use is growing in scanning probe microscopy. Nevertheless, only a few studies on soft biological samples have been reported using these probes. In this work, we present the methodology to develop and use these nanosensors to properly work with biological samples. The working principles, fabrication and experimental setup are presented. The results in the nanocharacterization of different samples in different ambients are presented by using different working modes: amplitude modulation with and without the use of a Phase-Locked Loop (PLL) and frequency modulation. *Pseudomonas aeruginosa* bacteria are imaged in nitrogen using amplitude modulation. Microcontact printed antibodies are imaged in buffer using amplitude modulation with a PLL. Finally, metastatic cells are imaged in air using frequency modulation.

## 1. Introduction

It was about 20 years ago that the first biological sample was imaged in liquid using AFM: the clotting process of fibrinogen upon activation with thrombin [1]. Since then, atomic force microscopy has become a highly valuable research tool in biology and medicine. Biological samples studied with AFM range nowadays from the smallest biomolecules (such as lipids [2], proteins [3], DNA [4], RNA) to subcellular structures, cells and tissues [5]. There are several main applications of AFM in cell biology: imaging, material properties measurements, binding force measurements and manipulation (see [6] for a recent review). The most direct application is imaging, and a large number of cells have been imaged in physiological buffer [7,8]. The key to imaging living cells is to couple the AFM with controlled culture systems. Controlling the temperature and the culture medium conditions allows one to image living cells over long periods of time [9,10]. Mammalian [11] and endothelial [12] cells, fibroblasts [13] or cardyocites [14] are just a few examples of the wide variety of cells with reported AFM imaging in buffer, but experiments in molecular and cell biology often require more than morphological data; they require the measurement of other properties of the sample to complement the topographic information. Some groups have introduced the concept of “cell surgery” based on AFM. In [15], the surface of a bacteria has been modified by using a functionalized AFM probe. In terms of cell manipulation, the localization and extraction of RNA from the cell by using an AFM tip have been done in [16], but standard AFM probes have some limitations, so efforts have been made in developing novel nanotools such as nanoneedles [17].

The AFM technique is based on the measurement of the interaction force between a nanometric radius tip and the sample surface. The tip is usually at the end of a microfabricated cantilever which bends when a force occurs. A laser is used to measure the deflection of the cantilever, which can limit the application of the technique under certain conditions (for example in multiprobe experiments, when working with samples with difficult access, or when there is a need to combine AFM with some optical microscopy technique). An alternative method which is being increasing in popularity is the use of a quartz tuning fork (QTF) probe. Originally introduced to scan sample surfaces with micrometer lateral resolution [18], QTFs have shown their ability to image the sample surface with nanometric accuracy [19,20]. A tip is mounted in the QTF resonator and oscillated parallel to the sample surface. The force interactions produce changes in the amplitude of oscillation and the resonant frequency of the probe; those signals can be used for feedback in topographic measurements.

The most direct benefit of using QTF sensors is their high quality factor when operating in liquids: just the fiber end is immersed in the liquid cell, so the *Q* can have a value up to 400–500. These values are more than one order of magnitude higher than typical values obtained in liquids with standard AFM cantilevers. Besides, the high static spring constant allows very low oscillation amplitudes, which together with the high *Q* provides a high sensitivity in the detection of the frequency shift and interaction forces. There have been reported in [21,22] that femtoNewton forces can be measured using these sensors in vacuum environment. In air and liquid environments, the sensitivity drops to the range of hundreds of picoNewtons [23]. Also, QTFs are sensors very stable towards temperature variations and they have very low energy dissipation when compared to a cantilever, so the force measurements are stable enough to work with biological samples.

Advantages in the potential applications are wide due to the fact that there is no need to use a laser and a photodiode to measure the interaction. The most direct is the combination with optical techniques [24]. Also, the tip is millimeters long, so sample access is much better than with standard cantilevers, and QTF experiments can be performed in Petri dishes, well-plates and other array-like sample preparations. Long tips and laser-free detection also facilitate the integration of more than one sensor in the measurement setup to conduct complex experiments with more than one probe [25,26]. Even the integration of QTF sensors in microanalysis systems and lab-on-a-chip devices could solve some of the problems related with mass detection using micromachined cantilevers.

The use of QTF to work with biological samples has not been extensively reported if compared with studies reported using conventional AFM cantilevers. Nevertheless, in the last few years some works have reported the use of QTF to image cells [27] and biomolecules [28], and for molecular recognition experiments [23,29], so the use of QTF for biological studies is increasing in popularity, but there is still a lack of information about protocols, benefits and drawbacks. In this work, we present the methodology to fabricate and use QTF for biological samples imaging, and some results obtained under different conditions (nitrogen, ambient and buffer) with different samples (bacteria, cells and biomolecules).

## 2. Material and Method

### 2.1. Quartz Tuning Fork Working Principle

Quartz tuning fork devices are mostly used in precise oscillation circuitry (e.g., in watches). The resonant frequency *f* of the device depends on the spring constant *K* and effective mass *m* of the tuning fork [30]:

(1) $$f=\frac{1}{2\pi }\sqrt{\frac{K}{m}}$$

Then, if a force *F* is applied to the fork, there will be a resonant frequency shift (that can be seen as a change in the effective mass or a change in the spring constant) which depends on the gradient of the force in the oscillation direction. For a harmonic oscillation model where both tines are oscillating, the frequency shift is [31]:

(2) $$\Delta f=\frac{f}{4K}\frac{\partial F}{\partial x}$$

The expression differs by a factor of 1/2 from the result obtained for conventional cantilevers; this factor reflects the fact that only one prong of the fork senses the interaction but both prongs are oscillating. Nevertheless, for a QTF, the effective spring constant is the double of the *K* for a cantilever beam, as reported in [32,33].

Then, the force sensitivity is related to the resolution in the frequency shift measurement. With the high *Q* factors of quartz tuning fork in liquid, the frequency measurement bandwidth is very narrow and the frequency shifts can be measured with high resolution [34]. Force sensitivity can be down to 0.2 pN/nm in vacuum [22], and forces of hundreds of picoNewton can be effectively measured with the tip immersed in liquid [23]. The sensitivity of the nanotools is, then, comparable with the most sensitive commercial cantilevers.

The tip mounted on the tuning fork nanotools are usually sharpened wires or fibers attached to one of the tines of the device. There are two main configurations depending if the oscillation is made parallel (shear mode) or perpendicular (normal oscillation mode) to the surface.

If operated perpendicular to the surface, the interaction force reduces the amplitude of oscillation, and some numerical methods have been implemented to calculate the interaction normal force from this amplitude reduction measurement [35,36]. If operated parallel to the surface, when the tip approaches to the sample surface, both friction (*F_f_*) and elastic (*F_e_*) forces appear:

(3) $$\begin{array}{l} F_{f}=M\gamma \dot{x}\\ F_{e}=kx \end{array}$$

where *γ* is the damping coefficient and k is the tip-sample interaction constant, defined in [37] as:

(4) $$k=\left[{\left(\frac{\Delta f}{f}\right)}^{2}-1\right]K$$

The technique used to measure this oscillation depends on the solution used to drive the tuning fork to its mechanical resonant frequency. There are two main alternatives:

1. The QTF is driven mechanically by a dither piezo. Then, using a charge amplifier the piezoelectric voltage generated can be electrically measured.
2. The QTF is driven electrically and the oscillation amplitude is measured by using a current-voltage converter.

For the mechanically driven, the device should be calibrated to relate the measured voltage with the amplitude of oscillation. For the electrically driven device (with a signal of amplitude *V_rms_* at frequency *f*), the amplitude of oscillation *A* can be evaluated from the current *I_rms_* by using the model in [38]:

(5) $$A=\sqrt{\frac{Q\cdot V_{\text{rms}}\cdot I_{\text{rms}}}{K\cdot 2\pi f}}$$

**Figure 1.**
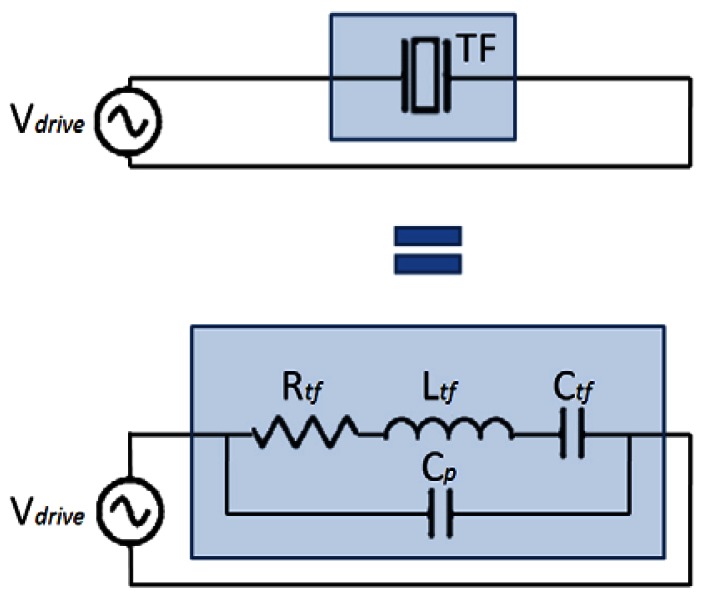
Butterworth-Van Dyke equivalent circuit for the tuning fork.

From the electrical point of view, piezoelectric oscillators can be modeled by the Butterworth-Van Dyke equivalent circuit [39], as shown in Figure 1. The capacitance and inductance model the potential and kinetic energy storage respectively, the resistor models the dissipation and the parallel capacitance models the parasitic capacitor due to the electrodes.

### 2.2. Probes Fabrication

**Figure 2.**
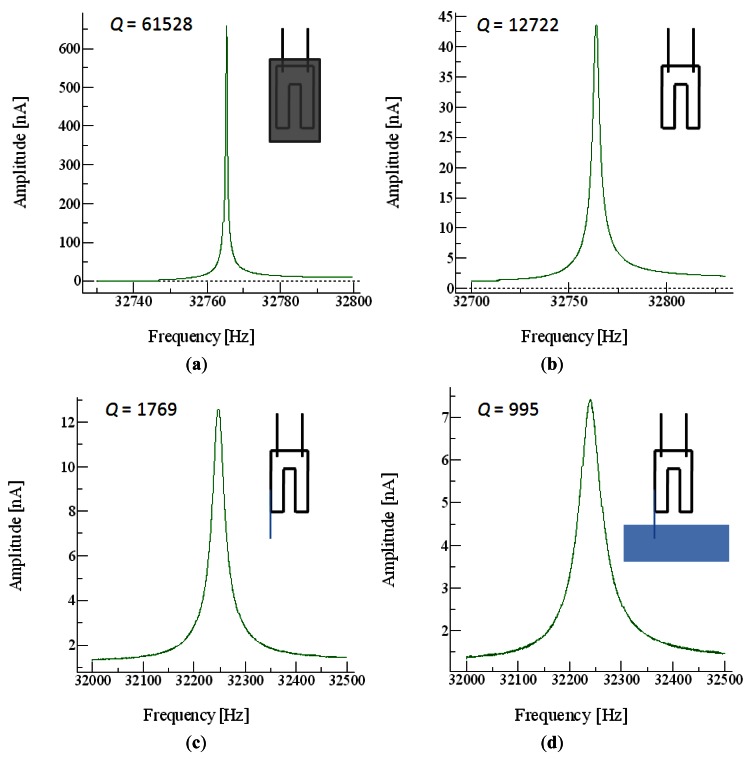
(**a**) *Q* is 61,528 in vacuum and (**b**) 12,722 when resonating in air without the attached fiber; (**c**) Once the fiber is attached, *Q* is 1,769 in air and (**d**) 995 when the tip is immersed in liquid.

QTF devices are extremely good mechanical resonators, with high quality factors (*Q*, the relation between the resonant frequency and the bandwidth of the device) of up to 40,000 when operated in a vacuum. In air, the quality of the resonance is still so high that a probe can be glued to a tine to act as the end-effector of the nanotool. Even when the end-effector is introduced in liquid, the *Q* factor is still high enough to allow good images to be acquired using the QTF (Figure 2).

**Figure 3.**
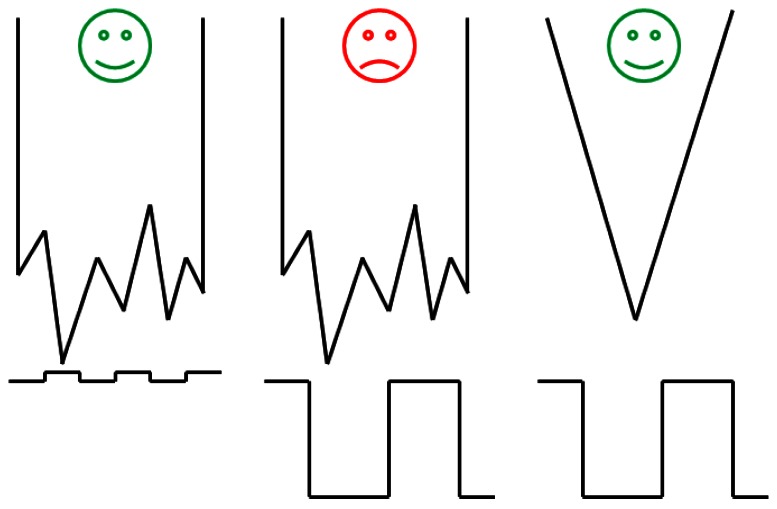
Different situations where the fiber can be just broken (for “flat” surfaces) or must be sharp (“non-flat” surfaces).

The fibers attached to the tuning fork had a diameter of 125 μm. For the inspection of very flat surfaces (a few nm of roughness in μm-sized areas) a simple solution was to break the fiber and measure the interaction between the most external peak of the fiber and the surface. But for non-flat surfaces this was not possible (Figure 3).

**Figure 4.**
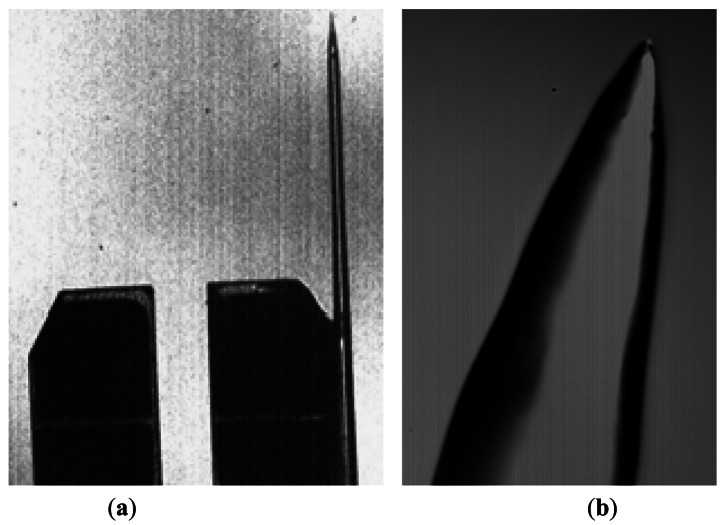
(**a**) Photograph of the QTF probe; (**b**) Optical microscope image of the sharpened fiber end.

So to devise the probe, a 32.768-kHz quartz tuning fork (model AB38T from ABRACON Corp.) resonator was decapsulated. Then, 125-μm SiO_2_ fiber was chemically sharpened as proposed in [40] and glued to one of the tines of the tuning fork (Figure 4).

Briefly, the fiber was immersed into a 40% HF solution with a protective isooctane (C_8_H_18_) layer; then, in the interface between the acid and the protective layer, a meniscus was formed. As the radius of the fiber was decreasing due to the HF etching, the radius of the meniscus progressively decreased. Finally, the tip was full immersed in the isooctane and the process auto-stopped.

The length of the part of the fiber protruding from the fork was between 3–4 mm, which was the minimum length to work with the liquid cell while maintaining the QTF resonating in air. The longer the fiber, the lower of the *Q* obtained due to the extra mass and unbalancement of the sensor. Anyway, more important for the drop of the *Q* is the immersion depth of the fiber end in the liquid [41].

The resolution in the *X* and *Y* directions achieved with the sensor was determined by the tip radius and cone angle. To measure these parameters, specific microfabricated samples were scanned with the sensor. Tip radius was of the order of 300 nm and the cone angle was 45°.

### 2.3. Electronics

**Figure 5.**
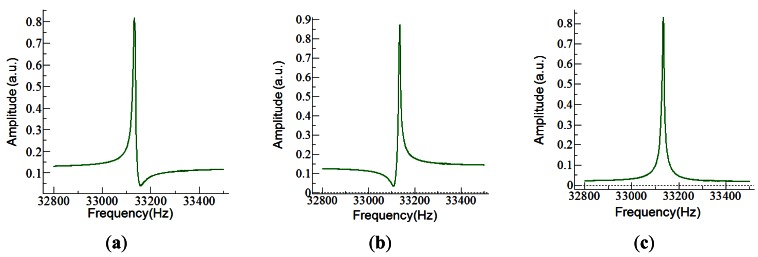
Different situations when compensating the parasitic capacitor with the developed circuitry: undercompensated (**a**), overcompensated (**b**), and adjusted (**c**).

The easiest way to drive the tuning fork and measure the current was to use a simple circuit with a transimpedance amplifier (TIA). The choice for the TIA was an OPA656 from Texas Instruments, with a 10^6^ V/A gain. For these gain, the amplifier presents a 1 MHz bandwidth, high enough for working with the QTF. The main problem with this solution was the current flowing through the parasitic capacitor which became dominant away from the resonant frequency of the fork; it was responsible of asymmetries (Figure 5) and shifts in the frequency response, and it limited the signal-to-noise ratio (SNR).

The current through the fork (*I_tf_*) and through the parasitic capacitor (*I_p_*) with a simple driving circuit are given by Equations (6,7). The SNR of the system is frequency-dependent and limited by the parasitic capacitance (*C_p_*) as shown in Equation (8):

(6) $$I_{p}=V_{\text{drive}}\cdot j\omega C_{p}$$

(7) $$I_{tf}=\frac{V_{\text{drive}}}{R_{tf}+\frac{1}{j\omega C_{tf}}+j\omega L_{tf}}$$

(8) $$\text{SNR}=20\log\left(\frac{I_{tf}}{I_{p}}\right)=20\log\left(\frac{1}{C_{p}(L_{tf}\omega^{2}+R_{tf}j\omega +1/C_{tf})}\right)$$

**Figure 6.**
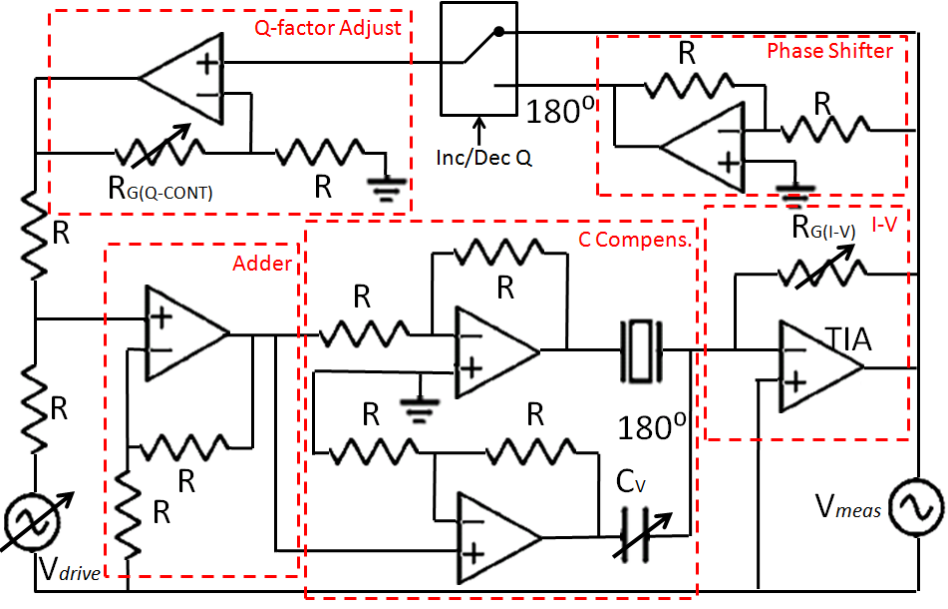
Scheme of the electronic driver. Feedback is made in the current through the fork (in-phase to increase *Q* and counter-phase to lower *Q*).

To maximize SNR, *C_p_* was effectively made null in the frequencies near the resonance frequency of the tuning fork. To this end, a capacitor-compensated circuit was implemented to drive the fork (Figure 6); *I_p_* was compensated with a subcircuit with the same capacitance but 180° phase shifted [42]. Consequently, only the current through the fork was being amplified by the TIA.

Once *C_p_* was compensated for and SNR maximized, the critical parameters of the sensor were *A* and *Q*. The *Q* of the sensors differed depending on the fabrication conditions. So the implemented driver integrated an analog *Q*-controller. The current through the fork was measured by the TIA and a feedback signal (in-phase to increase the *Q* factor and counter-phase to decrease the *Q* factor) was added to the fork's driving voltage. The gain of the *Q*-control was adjusted with an external potentiometer. A signal was added in-phase or in counter-phase to the QTF driving voltage to increase or decrease the *Q* factor of the device.

The role of increasing or decreasing the *Q* is still controversial in the literature. It is well known that lowering the *Q* increases the measurement bandwidth, and the imaging scan speed can be increased as well [43]. But the improvement accomplished by increasing the *Q* electronically is still not clear. Some works have shown that imaging the samples by increasing the *Q* reduces the interaction (increases the sensitivity) and the height observed in the samples is more accurate [44], but other works report that no effective improvement is achieved by using the *Q*-control, because the improvement in sensitivity is counteracted by the increased thermal noise [45].

### 2.4. Imaging Modes

**Figure 7.**
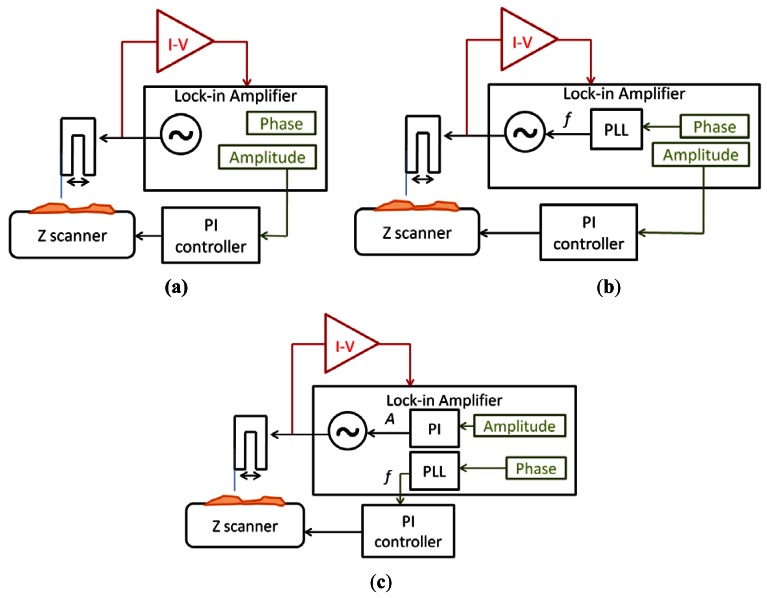
(**a**) Scheme of the amplitude modulation mode; (**b**) Amplitude modulation with a PLL to track the resonant frequency; (**c**) Scheme of the frequency modulation mode.

The signal to drive the QTF probe was generated by an integrated generator in the lock-in amplifier (Nanotec Electrónica S.L. dynamic board). Then, the current was measured by the I-V converter (TIA) and used as input of the lock-in amplifier. The measured signal had two different information about the interaction between the tip and the sample: the amplitude and the phase. Depending on the use of these signals, three different imaging modes were implemented:

- Amplitude modulation (Figure 7(a))
- Amplitude modulation with a PLL (Figure 7(b))
- Frequency modulation (Figure 7(c))

The simplest mode was amplitude modulation. The amplitude of the current was used as main feedback for the Z movements of the scanner. A lower amplitude than the free oscillation amplitude was imposed to the system and the sample was moved in Z direction to maintain constant this amplitude.

An extension of the amplitude modulation mode was to use a Phase-Locked-Loop (PLL) to maintain the sensor in resonance. The PLL changed the frequency of the excitation signal to maintain a constant 0 phase, while the amplitude was still used as main feedback for the topography imaging. This method was suitable for working in liquids, because the resonant frequency of the sensor changed with the evaporation of the buffer (the resonant frequency depends on the relation between the length of the fiber immersed in the liquid and the part of the sensors resonating in air). We didn't use any fluidic compensation for the experiments in buffer, so the frequency changed while the evaporation occurred and the only way to compensate this effect was to use a PLL to track the resonant frequency.

Finally, the third mode was frequency modulation. The main feedback (to reconstruct the topography) was made in the frequency of the sensor, while a PI feedback was made in the amplitude of the driving signal to maintain constant the amplitude of oscillation. This mode was more noisy because of the secondary feedback, and the bandwidth was limited, but the control over the interaction was greater than in amplitude modulation: frequency modulation mode was more adequate when the sample was very adhesive and we wanted to assure that the interaction was conservative and no friction forces appear. A similar method, but without PLL, is to use Phase Modulation, where the main feedback is made in the phase directly. This method is less noisy (due to the noise introduced by the PLL) but the frequency drift (especially in liquids) make difficult its use because the measurement bandwidth is very narrow.

## 3. Results

### 3.1. Imaging Bacteria in Nitrogen Using Amplitude Modulation

**Figure 8.**
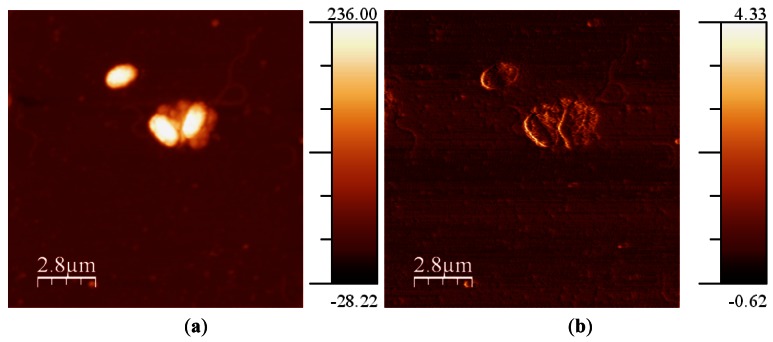

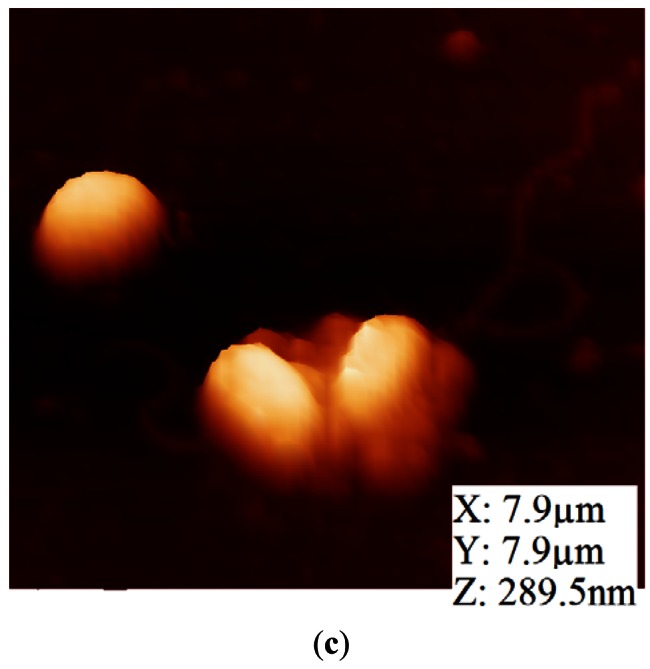
(**a**) Topography image of bacteria acquired in amplitude modulation (Z scale in nanometer); (**b**) Error image (amplitude, Z scale in nA); (**c**) 3D representation.

*Pseudomonas aeruginosa* bacteria were grown over flat gold surfaces without any specific fixation. Then, the samples were dried and imaged in nitrogen ambient. The QTF sensor was oscillated 1 nm and its *Q* was selected (by using the Q-controller to decrease it) to be 1,000. This *Q* was the minimum we were able to obtain with the sensor (originally with a *Q* greater than 3,000); it was decreased to allow the feedback controller to respond at an acceptable time. The amplitude setpoint was settled to the 95% of the free oscillation amplitude (Figure 8).

As the images were acquired in nitrogen, adhesion between the tip and the sample due to the meniscus forces was low. In the error image (amplitude), it can be seen that there was not a high difference between the bacteria and the substrate, so amplitude modulation was a good method to image bacteria under these conditions.

Bacteria were about 300 nm in height, but the flagella were only a few nanometers, so the images in frequency modulation would be very noisy for these samples. This is due to the fact that introducing a second feedback loop increases the noise measurement. Also, images should be acquired more slowly due to the decrease in the response time of the controller.

### 3.2. Imaging Antibodies in PBS Buffer Using Amplitude Modulation with a PLL

**Figure 9.**
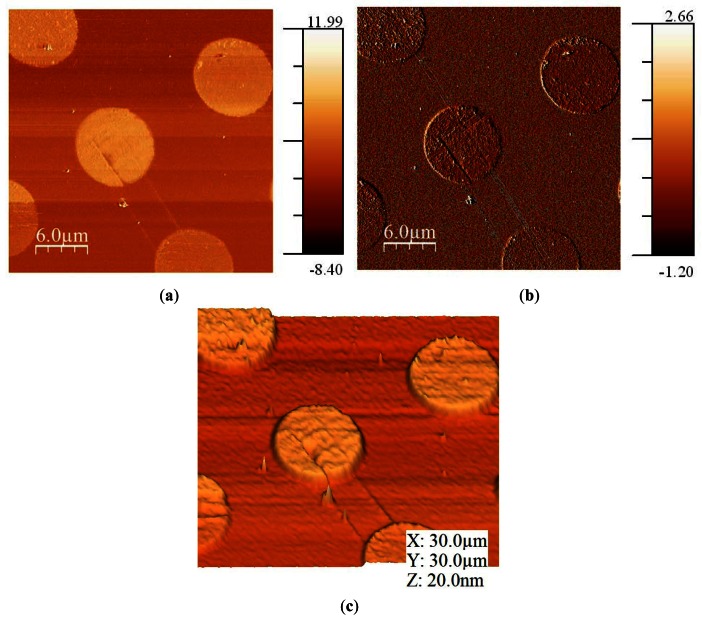
(**a**) Topography image of microcontact printed antibodies acquired in amplitude modulation with PLL (Z scale in nanometer); (**b**) Error image (amplitude, Z scale in nA); (**c**) 3D representation.

IgG antibodies were anchored onto gold substrates by a microcontact printing technique [46]. A pattern of alkylthiols with a spot diameter was 10 μm was used to immobilize the antibodies. Images were acquired in PBS buffer (pH = 7.4) with a QTF probe. The *Q* factor was increased from 120 to 380 by means of the Q-controller. It was the maximum *Q* value we were able to obtain with the sensor, to minimize the interaction force between the fiber tip and the antibodies layer. The PLL was used to maintain the sensor in resonance while the amplitude was used for the main feedback (Figure 9). The setpoint was settled to the 95% of the free oscillation amplitude.

These samples are usually acquired in friction mode with conventional AFM techniques, but the sensitivity of the QTF is enough to image these samples in amplitude modulation. The PLL was used to maintain constant the resonant frequency and to avoid as much as possible the drifts due to the evaporation of the buffer in the liquid cell (because the experiments lasted for hours). We didn't use any fluidic compensation, as the use of the PLL was enough to compensate the frequency drift due to the buffer evaporation.

Images showed that the mean height of the antibody layer was between 15 nm and 20 nm, and no friction was evidenced because of the poor contrast in the error image. The accepted dimensions for IgG are 8.5 nm × 14.4 nm × 4 nm as reported by [47]; microcontact printed IgG imaged by AFM have shown to have a height in concordance as presented in [48].

The height was greater than other studies over IgG antibodies done with QTF reported in the literature [28]; this could be to the fact that in the present experiment the antibodies were imaged in buffer (the morphology of the antibodies could be heavily affected by the drying process) and that the height measured is the antibody together with the alkylthiol layer. This method combines the lower noise of the Amplitude Modulation (due to the use of only one feedback loop) with the stability in liquid given by the PLL, so it was the most suitable to image these kind of samples in buffer.

### 3.3. Imaging Cells in Air Using Frequency Modulation

**Figure 10.**
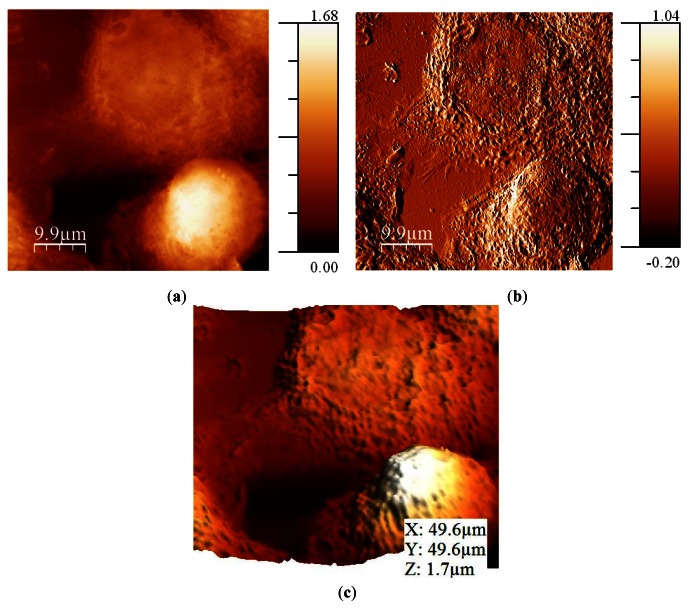
(**a**) Topography image of the cells acquired in frequency modulation (Z scale in micrometers); **(b**) Error image (frequency, Z scale in Hz); (**c**) 3D representation.

Breast metastatic cells (MDA-MB-468) were prepared over a collagen-covered glass cover. These cells do not naturally reside on glass in their native environment, so the collagen coating was used to avoid changes in the morphology induced by the substrate. After that, cells were fixed using a 4% formaldehyde treatment. It is possible to image cells in air without fixation if the experiment is performed within the first 10 minutes [49], but experiments usually lasted some hours. Also, fixing the cells avoided potential structural changes caused by drying forces [50]. Amplitude was maintained constant at 5 nm and the *Q* was fixed to 620. *Q* needed to be as low as possible because cells had a high in the range of microns and the controller needed to respond quickly to avoid sample damage. The frequency shift imposed was 6 Hz (Figure 10). In the error image (frequency shift), it can be observed that the interaction was settled low, so there was much information in this image. It was due to the high adhesion between the tip and the cells and the collagen, and the need to not damage the cell while imaging.

## 4. Conclusions

In this work, the methodology to image biological samples with QTF probes is presented. Three different samples in different environments are chosen. Then, the most adequate method is used to image each of them with the nanosensors. Results show that the QTF can be a good alternative to standard AFM cantilevers. It presents some advantages such as the mm-sized tip and the elimination of the laser-photodiode measurement system.

The main drawbacks of QTF probes are that they are usually home-made and that there is not a clear methodology to work with them. We overcome the first problem by adjusting the quality factor of the device to have the same dynamical response with different sensors [51]. With respect to the methodology, working in amplitude modulation has more SNR (because of the added noise of the second loop introduce by the PLL) and it is appropriated when there is no a heavy adhesion and the frictional force is not important. In liquid or with high-adhesive samples, tracking of the frequency is needed: there is an important problem with the evaporation when working in liquid and a loop in the frequency is needed. With highly adhesive samples, the need to minimize the friction forces makes the frequency modulation method the most suitable one, even with the sacrifice of the resolution because noise is increased by the second feedback loop.
